# The Elements of Executive Attention in Top Soccer Referees and Assistant Referees

**DOI:** 10.2478/hukin-2014-0025

**Published:** 2014-04-09

**Authors:** Przemysław Pietraszewski, Robert Roczniok, Anna Maszczyk, Paweł Grycmann, Tomasz Roleder, Arkadiusz Stanula, Olga Fidos - Czuba, Marcin Ponczek

**Affiliations:** 1 Department of Sports Theory, Jerzy Kukuczka Academy of Physical Education in Katowice, Poland.; 2 Department of Finance and Insurance, University of Economics in Katowice, Poland.; 3 Department of Team Sports, Jerzy Kukuczka Academy of Physical Education in Katowice, Poland; 4 Department of Cardiology Medical University of Silesia, Katowice, Poland.; 5 Department of Psychology, Jerzy Kukuczka Academy of Physical Education in Katowice, Poland.

**Keywords:** soccer referee, perceptual skills, psychomotor abilities

## Abstract

The aim of the present study was to compare executive attention of top soccer referees and assistant referees at different levels of professional attainment. The sample consisted of 53 subjects (FIFA and national level) – 30 referees and 23 assistant referees. Executive attention of assistant referees was significantly better than the referees’ (p<0.01). Furthermore, extraclass and international referees demonstrated better executive attention than the first-league referees (p<0.01). The research results have proved that referees’ executive attention differs depending on their function and professional level, as well as indicated that the quality of abilities may influence the number and correctness of decisions made during a game. This elementary cognitive process may be strongly shaped by individual’s experience and age. This finding may be instrumental in screening referees and developing criteria for recruiting future referees.

## Introduction

The large number of stimuli likely to affect referee’s decisions requires increased concentration as well as the reception and processing of information coming from many sources, while maintaining the necessary speed and precision of reactions. To measure up to the standards, referees must have special skills (MacMahon et al., 2006). Having to cope with tactical tasks and to deal with situations as they occur during a game, a soccer referee must work at a rate dictated by external circumstances, such as the speed of the game and the teams’ tactics. They must also consider the players’ technical skills, the atmosphere on the pitch, in the technical areas and in the stand, the profiles of particular players and many others operating in the immediate environment. It is quite obvious that the appropriate physical preparation is a factor enabling referees to absorb and process this multitude of information. This aspect has been explored by many authors (Castagna et al., 2002; Impellizzeri et al., 2005; Krustrup and Bangsbo, 2001; Mallo et al., 2007; Weston et al., 2006). Less than twenty years ago decisions about referee’s suitability for officiating a game at a given level were mainly made in relation to different aspects of visual abilities, in addition to the assessment of physical conditioning. The examined abilities were relatively simple, e.g. visual acuity, colour perception, or response time to various visual stimuli. Sometimes more complex functions, such as visual-motor coordination, were assessed as well. The tests performed today are more comprehensive, involving for instance the simulation of real tasks that referees have to handle on the pitch. The recently introduced procedures and tests address cognitive processes, which are extremely important psychological mechanisms determining the performance of referees and assistant referees. Some studies have attempted to establish the influence of physical and perceptual-cognitive abilities on the performance of assistant referees and their errors (Catteeuw et al., 2010a), while others sought new tools for increasing the efficiency of decision-making training for referees (Helsen and Bultynck, 2004). This trend stems from the fact that the psychomotor abilities constitute key determinants of individual’s usefulness as a referee. The abilities are determined by the level of cognitive processes (Catteeuw et al., 2010b). Generally, perception is a human faculty responsible for receiving information from the environment. It is composed of different sense modalities, such as vision, hearing, taste, smell, touch, sense of equilibrium, etc. This mental process involves stronger concentration of cognitive processes on an object (a task) or increased awareness of the operating stimuli (Strelau, 2000). In a game situation, perception allows reacting to suddenly appearing stimuli, such as movement against a motionless background or stillness against a moving background, a sudden change in direction, etc. Cognitive processes that a situation triggers in the referee independently of their will are called involuntary. They are activated when the referee instinctively and involuntarily directs their faculties to the source of the stimuli. Another situation enhancing referee’s cognitive processes (perception) is voluntary concentration on the task. Both types of perception accompany referees in all their activities throughout the game.

An integral element of perception is attention that in some situations may be treated as a perceptual process in its own right. Attention is a tool that allows the referee to select the received information. It is characterised by:

- A level of concentration expressed by a limited range of objects selected to receive attention.
- A range of attention understood as the number of objects or object’s characteristics that can be included in the range of attention and be perceived at the same time.
- Divisibility of attention, which is the ability to divide attention between two or more activities.
- Alternation of attention, which allows the focus of attention to be quickly switched between objects or people.
- Sustainability of attention, i.e. the ability to maintain attention on an object or activity for a longer time.

Expert soccer referees must have good visual skills to make good decisions quickly and accurately in different situations (Ghasemi et al., 2009). Such skilled behaviours require many years of practice, together with a considerable amount of ability (Williams and Ericsson, 2005). It is now accepted that successful performance in this sport requires skill in perception (Williams and Hodges, 2005a). An inadequate level of the properties increases the probability of error. There is a whole range of studies in the contemporary literature which explore the sources of referees’ errors, such as misjudgement of the offside line (Catteeuw et al., 2010, 2010a; Gilis et al., 2009; Helsen et al., 2006; Krustrup et al., 2002). The referee sometimes receives a significant amount of information from several senses. It is therefore quite obvious that perceptual efficiency is crucial in the first stage of decision-making, when the most important pieces of information are absorbed and sorted out. In analysing a game situation, the referee considers both information that they can verify by themselves, as well as external information received from other members of the referee crew via the radio and electronic flags system (the BIP). Given that a referee makes an average of 140 decisions during a game, all likely to affect its final result, the ability to receive much data from different sources and to sort them out appropriately becomes even more important (Helsen and Bultynck, 2004). The referee must be particularly attentive for at least 90 minutes of the game, but sometimes this requirement extends beyond 120 minutes. When the level of fatigue increases, the referee must be extremely focused to be able to handle disputable situations. This is especially obvious in modern soccer which has become a very fast, dynamic, technical and tactically complex sport. This study aimed to compare the executive attention of top soccer referees and assistant referees at different levels of professional attainment.

## Material and Methods

### Participants

The study involved 30 referees and 23 assistant referees combining a sample of 53 subjects. Thirteen of them were international FIFA referees (6 referees and 7 assistant referees), 29 were Extraclass referees (13 referees and 16 assistant referees) and 11 represented the first-league level (only referees). The referees were aged 32.7 years ± 3.7 years, their mean body height was 182.6 ± 4.6 cm and mean body mass 80 ± 15.6 kg. The respective characteristics of assistant referees were as follows: age 34.9 ± 3.5 years, body height 181.8 ± 5.7 cm, body mass 81.1 ± 7.7 kg. By the level of professional achievement, the descriptive statistics were the following: international referees – mean age 36.6 ± 4.2 years, mean body height 180 ± 4.2 cm, mean body mass 79 ± 13.3 kg; Extraclass referees – mean age 33.6 ± 2.5 years, mean body height 181 ± 5.5cm, mean body mass 81 ± 8 kg; first-league referees – mean age 31 ± 3.3 years, mean body height 183 ± 6.3 cm, mean body mass 81 ± 13 kg. All participants were informed about the research procedures and purpose of the study and were tested under identical circumstances. This project was approved by the Bioethics Committee for Scientific Research at the Academy of Physical Education in Katowice.

### Measures

The Toulouse-Pieron test is a classical attention test. The instructions are very simple, so that the participant’s concentration of energy and resistance to disturbances can be tested without interference from factors such as the level of intelligence, reading speed, etc. The participant is asked to mark on the test form, as fast and accurately as possible, symbols corresponding to the model. The testing procedure employed in this study used squares with a line attached to one of their corners. The participants were instructed to examine the symbols line by line from left to right and to mark those matching two model symbols within 5 minutes. The test produced three indicators – the speed of performance (SP) measured by the total number of squares reviewed and marked within 5 minutes, both correct and incorrect; the number of errors (NE) represented by the number of incorrectly marked symbols or correct symbols missed; the correct answers (CA), i.e. the number of correctly marked symbols. With this information, the main parameter called the Precision Index (PI) was calculated as a percentage rate using the following formula PI = (CA – NE) × 100 / CA. The test, informally known as „Spiky Squares‟, has been used in a variety of studies and emerges as both valid and reliable for the specific samples used in these studies (Gomes et al., 1999; López and Vazques, 2003). The participants were informed, that the results of all tests may affect the recruitment of the refereeing games in the season, so they were well motivated.

### Statistical Analysis

The referees and assistant referee data were compared using an unpaired t-test with Bonferoni corrections. The one way analysis of variance (ANOVA) was used to test referees and assistant referees for statistically significant differences. Then the first-league referees, the Extraclass referees and the international referees were tested to find out if they were significantly different. Statistical significance was set at p<0.05 for all statistical tests. A Tuckey’s post-hoc procedure was used to make further evaluations of any significant results obtained from ANOVA. Correlation coefficients were determined and tested for significance using the Pearson’s regression test. All statistical analyses were performed with the STATISTICA package, version 10.0 (StatSoft Inc., Poland). The data are presented as means ± standard deviation, unless otherwise stated.

## Results

Table 1 presents the mean age, body height, body mass, experience and the results of the Toulouse-Piéron attention test taken by the participants. Significant differences were found for the mean values of the Precision Index (t= −3.98; p=0.0003). The assistant referees performed better in the test than the referees. Significant differences were also found for the variable ‘number of errors’(t= −4.24; p=0.0001). The referees committed more errors than the assistant referees. The referees and the assistant referees were also different taking into consideration age – the latter were significantly older (t= −2.45; p=0.03). As far as other variables are concerned, differences were not found.

A one-way ANOVA was performed to reveal whether the differences between the values of the examined variables obtained for all participants (first-league, Extraclass and international referees) were statistically significant. The first-league, Extraclass and international referees were found to differ with regard to the Precision Index (F=11.02, p=0.0001). In analysing the results of the post-hoc tests statistically significant differences were observed between the first-league referees and the Extraclass referees (t= −3.45; p=0.0008) and between the first-league referees and the international referees (t= −2.53; p=0.01), threfore the Extraclass referees and the international referees were not significantly different (t= 0.36; p=0.64).

The ‘Number of errors’ was another variable to reveal differences (F=8.87, p=0.0004). The results of the applied post-hoc tests indicated significant differences between the first-league referees and the Extraclass referees (t= −3.07; p=0.002) and between the first-league referees and the international referees (t= −2.13; p=0.023). The Extraclass referees and the international referees were not found to differ from each other (t= 0.39; p=0.65). The Extraclass referees ranked first both regarding the Precision Index and the number of errors, while the international referees and the first-league referees took second and third position, respectively.

**Figure 2.**
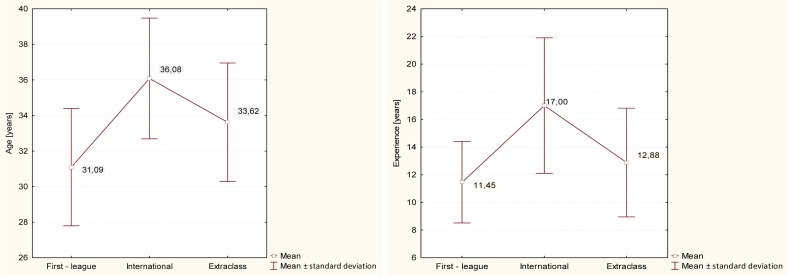
Analysis of variance for the variables Age and Experience

**Figure 3.**
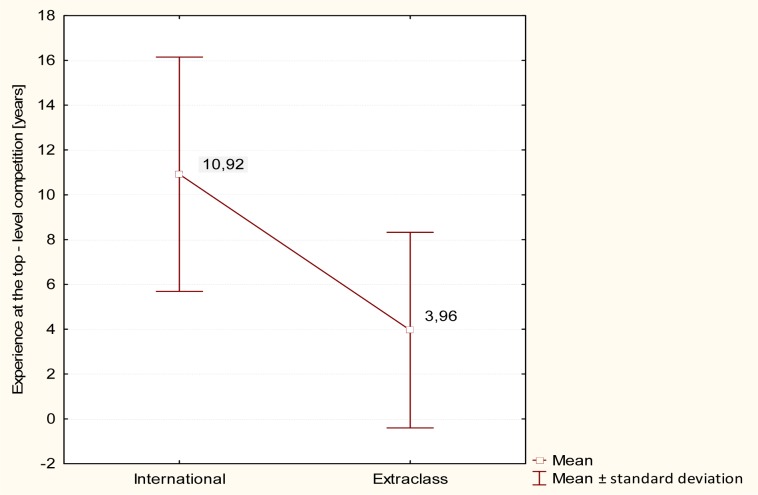
Analysis of variance for the variable Experience at the top – level competition

Significant differences were also found for such variables as age (F=6.39, p=0.003), experience (F=6.34, p=0.003), and years of officiating in the Extraclass league (F=18.21, p=0.0001). Taking into account age, only the first-league referees and the international referees who were the oldest were found to differ from each other (t= −2.98; p=0.002). These two groups were significantly different again with respect to another variable, i.e. ‘experience’ (t= −2.69; p=0.005), likewise the Extraclass referees and international referees (t= −1.78; p=0.04). The results of analysis are presented in Figures 2 and 3.

The next step in the research involved the same variables, but this time only referees without assistant referees were considered. The variance analysis revealed significant differences in the Precision Index (F=5.02, p=0.015) and its results were confirmed by post-hoc tests. Significant differences related to the Precision Index (t= −2.33; p=0.012) were only found between the first-league referees (86.97±4.15) and the Extraclass referees (92.02±3.91). The results of the Extraclass referees and the international referees (90.61±3.79, p=0.82) and the first-league and the international referees (t= −0.49; p=0.31) were not significantly different. This means that the weakest results were obtained by the first-league referees, who were outperformed by international referees and Extraclass referees who won the test. Another variable to reveal differences between its mean values after the one-way ANOVA was applied was age (F=5.22, p=0.013).

However, differences were only found between the first-league referees (31.09±3.3) and the international referees (36.6±4.21, t= −1.94; p=0.029). As expected, the variance analysis applied to referees’ experience displayed differences too (F=6,21; t= −2.62; p=0.006), but only between the first-league referees (11.45±2.94) and the international referees (17±3.43, t= −2.18; p=0.017).

In the next step, correlations between the anthropometric parameters, experience and the perception test results were estimated. Weak, positive, and significant correlations were only found between age and the Precision Index (r=0.34, p=0.019), and between age and the ‘number of errors’ (r=0.31, p=0.033).

## Discussion

The results provided by this study clearly show that referees’ executive attention vary depending on their function and the level of professional attainment, and that the quality of perception may influence the number and precision of decisions. They also demonstrate that referees’ experience and age may strongly determine their executive attention. This knowledge may be instrumental in screening referees and developing criteria for recruiting future referees. The aim of the study was to test and compare the executive attention of the top soccer referees and assistant referees and to find out whether relationships between the selected indicators can explain their values. The study’s results confirmed earlier conjectures that assistant referees have much better executive attention (PI = 94.13±3.85) than referees (PI = 89.62±4.36, p<0.01). This difference may be explained in terms of function-specific requirements. The study subjects were only different in the range of the tasks they were expected to fulfil (referee / assistant). Assistant referees frequently have to monitor many elements of the game to be able to assess the situation, such as foul play, the offside line, or kick-offs. This process requires full concentration and divisible attention (Catteeuw et al., 2010b). Moreover, unlike the lead referees, they cannot choose the optimal position for watching the situation, even though their position during the game determines whether the situation will be correctly assessed and, if inappropriate, may contribute to errors (Oudejans et al., 2000). In choosing their position assistant referees must comply with the rules and react to the situation on the pitch (the offside line). Interestingly, in this study referees performed only slightly faster (G718.6±97.9 - A686.7±86.3), but also made on average almost twice as many mistakes (G18.44±8.99 - A9.47±5.77 p<0.01). This means that assistant referees select stimuli definitely more efficiently, which may directly explain why they make fewer errors.

The variety of textbooks dealing with cognitive psychology proves that cognitive processes can be listed, classified and described from many angles, but this study concentrated on perception as a fundamental cognitive process. Given the complexity and variability of referees’ tasks, it is quite obvious that their actions demand full concentration (Catteeuw et al., 2010a). Referees have to respond to many perceptual and cognitive requirements (Helsen and Bultynck, 2004). Full concentration is particularly important in situations involving short-lasting, but very intensive physical and perceptual effort, such as following the counterattack that ends up with a foul in the penalty area or observing the offside line by assistant referees in dynamic situations. Even a temporary distraction of attention may result in misjudgement likely to distort the result of the game. Errors can be prevented by maintaining maximum mental concentration, i.e. by focusing all attention on the situation. The demands imposed on assistant referees are particularly high. There are many valuable studies on the special character of their tasks and the requirements they have to cope with (Catteeuw et al., 2009; Gilis et al., 2008; Mallo et al., 2008). The issue of assistant referees’ perceptual abilities has become so important that special training methods have been developed to improve them (Catteeuw et al., 2010a; Helsen et al., 2006).

Regarding the Precision Index, the first-league referees turned out to be definitely inferior to the Extraclass referees (t= −3.45; p=0.0008). They were also statistically less efficient than the international referees (t= −2.53; p=0.01). The Extraclass referees and the international referees were not statistically different from each other. The same pattern was found for the number of errors made in the test. The first-league referees were, again, less efficient than the Extraclass referees (t= −3.07; p=0.002) and the international referees (t= −2.13; p=0.023), but the Extraclass referees and the international referees were not different. Interestingly, the Extraclass-league referees ranked the highest for both the variables, whereas international referees were only second. The amount of information available at this stage of research was not sufficient to provide a reliable explanation to this ranking.

When referees were analysed alone, the number of statistically significant differences was definitely lower. It is interesting, though, that the Extraclass referees had the best results for the Precision Index again, but the first-league referees and the international referees were not different any more. That age-related (t= −2.98; p=0.002) and experience-related (t= −2.69; p=0.005) differences were found between the first-league referees and the international referees and that the first-league referees, the Extraclass referees and the international referees were also different for the Precision Index clearly shows that training and experience have a great impact on the development and improvement of some perceptual skills. In other words, long-term training may considerably correct perceptual deficiencies which affect referees in the first period of their careers, thus improving their performance. This means that age may positively contribute to the quality and adequacy of referees’ decisions. Similar conclusions have been drawn by researchers focusing on the impact of age on the physical preparation of English Premier League referees (Weston et al., 2010). It was demonstrated that although older referees run shorter distances during the game, they make their decisions as close to the ball and the site of the foul as their younger colleagues who run more, thus showing better skills of optimizing physical activity, which they probably acquire with experience. The impact of age on referees’ performance has also been the subject of other studies (Weston et al., 2006). Because the situation on the pitch is very changeable and the predictability of what will happen next is very low, referees must possess involuntary cognitive readiness and special abilities regarding concentration and divisibility of attention. Kosslyn et al. (1990) noticed that simple visual perception functions were accompanied by complex visual spatial perception processes. According to this concept, the absorption of visual information involves not only the pure perception of external changes (visual stimuli), but also activates the accumulated knowledge about their nature. Visual perception is constantly and dynamically enhanced by imagination and memory, anticipation of next events and abstraction, concentration of attention, as well as temporal cognitive processes (Kosslyn et al., 1990). The officiating of a game induces complex visual spatial perception processes in referees, who in extreme cases have to receive and process large amounts of information coming from different sources. Referees are expected to know which spot in the penalty area the crossed ball will hit, where frequently more than a dozen of players are struggling to take possession of it. Each physical contact may make the referee stop the game and penalize an individual player or the team. The referee must also be able to predict where the ball travelling with the given velocity and in the given direction, which he suddenly lost eye contact with, will reappear. The referee is also expected to know how the players of both teams may behave depending on their position on the pitch (Helsen et al., 2006). Important for these cognitive processes are the sense of passing time and the memory of temporal rhythms, but the ability to focus attention also plays an important role.

## Conclusions

The results of this study support role specificity in association football refereeing. Referees and assistant referees have a common goal: application of the Laws of the Game in a uniform and consistent way. However, even closely related roles such as those of referees and assistant referees require specific skills and abilities. The research results have proved that referees’ executive attention differs depending on their function and professional level, as well as indicated that the quality of the abilities may influence the number and correctness of decisions made during a game. Sport scientists and football governing bodies should acknowledge this when they produce development programmes for referees and assistant referees at different levels of professional attainment. This finding may be also instrumental in screening referees and developing criteria for recruiting future referees.

## Figures and Tables

**Figure 1.**
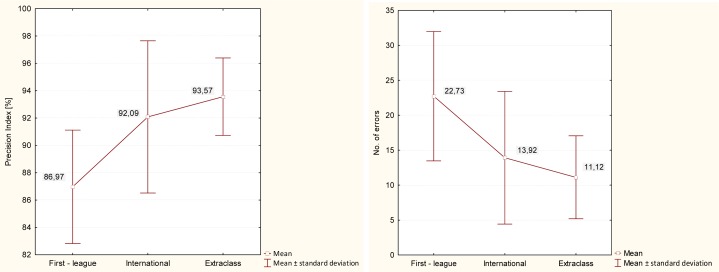
Analysis of variance for the variables Precision Index and Number of errors

**Table 1:** Anthropometric characteristics, experience and the results of the Toulouse-Pieron test for referees and assistant referees (mean ± SD)

Variables	Lead referees (n=30)	Assistant referees (n=23)
Precision Index (%)	89.62±4.36	94.13±3.85^**^
Speed of performance (n/5min)	718.6±97.9	686.7±86.3
Correct answers (n)	177.8±27.18	166.7±24.15
No. of errors	18.47±8.99	9.47±5.77^**^
Age (years)	32.73±3.69	34.89±3.55 ^*^
Body height (cm)	182.64±4.57	181.82±5.77
Body mass (kg)	80.4±15.65	81.16±7.73
Experience (years)	12.97±3.45	14.38±5.37
Experience at the top-level competition (years)	4.39±4.14	7.68±6.39
International experience (years)	5.27±3.91	8±5.24

significant differences

^*^p<0.05,

** p<0.01
